# Predictors of dizziness in older persons: a 10-year prospective cohort study in the community

**DOI:** 10.1186/1471-2318-14-133

**Published:** 2014-12-15

**Authors:** Otto R Maarsingh, Hanneke Stam, Peter M van de Ven, Natasja M van Schoor, Matthew J Ridd, Johannes C van der Wouden

**Affiliations:** Department of General Practice & Elderly Care Medicine, EMGO Institute for Health and Care Research, VU University Medical Center, Van der Boechorststraat 7, 1081 BT Amsterdam, The Netherlands; Cohort 6, International Primary Care Research Leadership Programme, University of Oxford, Oxford, UK; Department of Epidemiology and Biostatistics, EMGO Institute for Health and Care Research, VU University Medical Center, De Boelelaan 1118, 1081 HV Amsterdam, The Netherlands; Centre for Academic Primary Care, University of Bristol, Bristol, UK

**Keywords:** Older individuals, Dizziness, Predictors, Prospective cohort study

## Abstract

**Background:**

The current diagnosis-oriented approach of dizziness does not suit older patients. Often, it is difficult to identify a single underlying cause, and when a diagnosis is made, therapeutic options may be limited. Identification of predictors of dizziness may provide new leads for the management of dizziness in older patients. The aim of the present study was to investigate long-term predictors of regular dizziness in older persons.

**Methods:**

Population-based cohort study of 1,379 community-dwelling participants, aged ≥60 years, from the Longitudinal Aging Study Amsterdam (LASA). Regular dizziness was ascertained during face-to-face medical interviews during 7- and 10-year follow-up. We investigated 26 predictors at baseline from six domains: socio-demographic, medical history, medication, psychological, sensory, and balance/gait. We performed multivariate logistic regression analyses with presence of regular dizziness at 7- and 10-year follow-up as dependent variables. We assessed the performance of the models by calculating calibration and discrimination.

**Results:**

Predictors of regular dizziness at 7-year follow-up were living alone, history of dizziness, history of osteo/rheumatoid arthritis, use of nitrates, presence of anxiety or depression, impaired vision, and impaired function of lower extremities. Predictors of regular dizziness at 10-year follow-up were history of dizziness and impaired function of lower extremities. Both models showed good calibration (Hosmer-Lemeshow *P* value of 0.36 and 0.31, respectively) and acceptable discrimination (adjusted AUC after bootstrapping of 0.77 and 0.71).

**Conclusions:**

Dizziness in older age was predicted by multiple factors. A multifactorial approach, targeting potentially modifiable predictors (e.g., physical exercise for impaired function of lower extremities), may add to the current diagnosis-oriented approach.

**Electronic supplementary material:**

The online version of this article (doi:10.1186/1471-2318-14-133) contains supplementary material, which is available to authorized users.

## Background

The symptom dizziness is common among older persons: 30% of people above 65 years of age experience some form of dizziness, increasing to more than 50% in persons of 90 years and older [1, 2]. Dizziness can lead to severe limitations in daily functioning and is associated with depressive symptoms [3–5], poor self-rated health [2, 6], and reduced quality of life [7]. More importantly, it is a major risk factor for falling [7, 8], leading to fatal and non-fatal injuries and high healthcare costs [9, 10]. With the ageing of the population, the burden of dizziness – on society, health care systems, and individuals – will increase significantly.

Despite the aetiological differences between younger and older dizzy patients, guidelines on dizziness advocate the same diagnosis-oriented approach for all patients regardless of their age (http://cks.nice.org.uk/vertigo). However, this approach does not suit older patients presenting with dizziness. Often, it is difficult to identify an underlying cause in dizzy older patients. In 40% of dizzy older patients, general practitioners (GPs) record a symptom diagnosis as the final diagnosis (‘dizziness’ or ‘vertigo’) [11]. But even if an underlying disease is diagnosed, therapeutic options may be limited [5, 12]. Identification of long-term predictors of dizziness in older people may provide new leads for the management of dizziness in older patients.

The aim of the present study was to investigate long-term predictors of regular dizziness in persons above 60 years of age in a prospective cohort study with 7- and 10-year follow-up. We investigated predictors from six domains: socio-demographic, medical history, medication, psychological, sensory, and balance/gait, in a large nationally representative sample from the Longitudinal Aging Study Amsterdam (LASA).

## Methods

### Study sample

The study was conducted within LASA, an ongoing cohort study on physical, emotional, cognitive, and social functioning in older people in The Netherlands [13]. The LASA cohort was recruited in 1992 from a random sample of older men and women, aged 55–85, in the west, northeast, and south of The Netherlands. Since 1992, longitudinal data have been collected every three years. The sample was stratified by age, sex, degree of urbanization, and expected 5-year mortality. The sample is representative for the older Dutch population with respect to geographic region and degree of urbanization. Sampling, data collection, and nonresponse are described elsewhere [14, 15].

**Figure 1:**
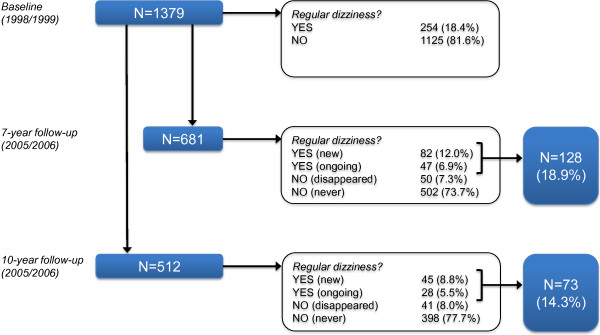
**Flowchart with dizziness prevalence at baseline, 7-year follow-up, and 10-year follow-up.**

The present study was performed among a subsample of the LASA cohort, consisting of 1,379 participants who were aged 60 years or older. This group of 1,379 participants answered a (face-to-face) question on dizziness during the third data collection cycle of LASA (1998/1999; Figure 1). We used the fifth and sixth data collection cycles of LASA (2005/2006 and 2008/2009) to conduct a 7-year and 10-year follow-up on dizziness. The 7-year follow-up sample included 681 participants with valid data at baseline on dizziness, the 10-year follow-up sample included 512 participants with valid data at baseline on dizziness (Figure 1).

Informed consent was obtained from all participants. The study was approved by the medical ethics committee of the VU University Medical Center.

### Ascertainment of dizziness

Dizziness was ascertained during face-to-face medical interviews in 1998/1999 (baseline measurement), 2005/2006 (7-year follow-up), and 2008/2009 (10-year follow-up). To assess dizziness, participants were asked if they were dizzy regularly (yes/no).

### Potential predictors of regular dizziness

Based on previous research, we examined 26 potential predictors of regular dizziness [2–5, 11, 16, 17]. Potential predictors were measured at baseline (1998/1999) and covered six domains: socio-demographic, medical history, medication, psychological, sensory, and balance/gait. The socio-demographic domain included age, gender, education, and household composition. Education was assessed as the highest level of education completed, ranging from 5 years (primary education) to 18 years (university). The domain medical history included history of dizziness and the self-reported number of major chronic diseases (0–7), history of chronic pulmonary disease, cardiac disease, peripheral arterial disease, diabetes mellitus, stroke, osteoarthritis or rheumatoid arthritis, and cancer. These self-reported answers correspond well with information from general practitioners [18]. The domain medication included the number of drugs used and the use of antidepressants, anxiolytics, hypnotics, antihypertensives, diuretics, or nitrates. Names of drugs were copied directly from the containers. The psychological domain included cognition, anxiety, and depression. Cognition was assessed by means of the Mini-Mental State Examination (MMSE; score of ≤24 as cutoff for cognitive impairment) [19]. Anxiety symptoms were measured using the general anxiety subscale of the Hospital Anxiety and Depression Scale (HADS-A; score 0–21) [20]. Depressive symptoms were measured using the Center for Epidemiological Studies Depression scale (CES-D; score 0–60) [21]. To improve clinical relevance, we used the dichotomous variable ‘presence of anxiety or depression’ (yes/no) with pre-established cutoff points for anxiety and depression (HADS-A score ≥11 or CES-D score ≥16) for the final analysis [20, 22]. The sensory domain included vision and hearing status. Visual and hearing impairment was assessed using two questions from the Organization for Economic Cooperation and Development long-term disability indicator [23], asking ‘can you see well enough?’ and ‘can you hear well enough?’ (yes/no). The domain balance/gait included gait speed (time needed to walk 3 meters, turn 180° and walk back as quickly as possible; test for functioning of lower extremities), chair stands (time needed to stand up and sit down five times with arms folded; test for functioning of lower extremities), tandem stand (ability to stand with one foot placed behind the other in straight line for at least 10 seconds; test for standing balance) [24], and fall history. We used dichotomous outcomes for gait speed (‘unable, or ≥10 seconds’; yes/no), chair stands (‘unable, or ≥15 seconds’; yes/no), and tandem stand (‘unable, or <10 seconds’; yes/no), with cutoff values based on previously determined categories in LASA (http://www.lasa-vu.nl/themes/physical/physicalperformance.htm). Fall history was assessed by asking ‘did you fall past year?’ (yes/no).

### Statistical analysis

The data were analysed in SPSS for Windows, version 20.0.0. In order to develop prediction models for regular dizziness at respectively 7- and 10-year follow-up, we performed multivariate logistic regression analysis with presence of regular dizziness at 7-year follow-up and 10-year follow-up as dependent variables. Prior to multivariate logistic regression analysis, we performed univariate logistic regression analysis to investigate associations between separate potential predictors at baseline and regular dizziness at 7- and 10-year follow-up. Variables were only entered in the multivariable regression model if the univariate *P* value was <0.1. In a backward elimination process (Wald test) we deleted variables from the initial model until only variables with a *P* value of less than 0.157 (Akaike Information Criterion) were retained in the final model [25]. We tested continuous variables for linear association with the outcome, which revealed no non-linear associations. We used Spearman’s rank correlation coefficient and the variance inflation factor to investigate multicollinearity. The number of major chronic diseases showed correlations of ρ > 0.5 with cardiac disease and osteo/rheumatoid arthritis, and was therefore not used in the final model. The number of used drugs showed a correlation of ρ > 0.5 with use of antihypertensives and was also not used in the final model. The multicollinearity analysis revealed no other relevant correlations between variables.

Because of the long-term follow-up and the risk to drop out, we compared characteristics of dropouts with non-dropouts at 7- and 10-year follow-up. Because some variables (n = 7) had missing values, we performed a missing value analysis. Because history of dizziness may be a strong predictor that overrules other predictors of regular dizziness [26], we also performed a multivariate regression analysis without history of dizziness as potential predictor.

### Performance of the models

To assess the reliability of the models we calculated the Hosmer-Lemeshow goodness-of-fit statistic. Calibration plots were constructed by first grouping respondents into categories based on the model-based predicted probabilities of suffering from regular dizziness at the end of follow-up. Categories used were 0-10%, 10-20%, …, 90-100%. The observed proportion of patients in each category was plotted against the center of the range defining the category (5%, 15%, …, 95%).

To assess the discriminative ability of the models we calculated the area under the receiver operating characteristic curve (AUC). Because prediction models perform better at the development cohort than in other similar populations, we used bootstrapping to adjust for over-optimism in model performance [27]. Bootstrap samples were drawn with replacement (n = 1000) from the two data sets (N = 681 and N = 512, respectively) and were used to compute adjusted AUCs. The variability of the adjusted AUCs over the bootstrap samples was quantified by the 2.5^th^ lower and 97.5^th^ upper percentile of the bootstrap distribution.

## Results

### Prevalence of dizziness

At baseline 254 out of 1379 participants reported regular dizziness (18.4%; Figure 1), 10.6% in those aged 60–69, 16.3% in those aged 70–79, and 26.5% in those aged 80 years or older. At 7-year follow-up 129 out of 681 participants reported regular dizziness (18.9%) and at 10-year follow-up 73 out of 512 (14.3%; Figure 1).

### Univariable associations with dizziness

Baseline characteristics significantly associated with regular dizziness at 7-year follow-up were gender, education, living alone, history of dizziness, history of peripheral arterial disease, number of drugs, use of anxiolytics, use of diuretics, use of nitrates, anxiety, depression, impaired vision, impaired functioning of lower extremities, and impaired standing balance (Table 1). Baseline characteristics significantly associated with regular dizziness at 10-year follow-up were living alone, history of dizziness, number of drugs, use of anxiolytics, anxiety, depression, impaired vision, impaired gait speed, and impaired chair stands (Table 1).

**Table 1:** **Univariable associations of baseline characteristics (1998/99) with regular dizziness at 7- and 10-year follow-up (2005/06 and 2008/09)**

	7-year follow-up (N = 681)					10-year follow-up (N = 512)				
Baseline characteristic	D+ (N = 129)	D- (N = 552)	OR	95% CI	*P*value	D+ (N = 73)	D- (N = 439)	OR	95% CI	*P*value
*I. Sociodemographic*										
Age, mean (SD)	74.0 (6.2)	72.9 (6.2)	1.0	1.0-1.1	0.052	72.4 (6.0)	72.0 (5.9)	1.0	1.0-1.1	0.609
Gender, female, n (%)	99 (76.7)	304 (55.1)	2.7	1.7-4.2	<0.001	48 (65.8)	253 (57.6)	1.4	0.8-2.4	0.193
Years of education, mean (SD)	8.3 (2.8)	9.4 (3.3)	0.9	0.8-1.0	0.001	9.0 (3.1)	9.4 (3.3)	1.0	0.9-1.0	0.315
Living alone, n(%)	67 (51.9)	182 (33.0)	2.2	1.5-3.2	<0.001	36 (49.3)	144 (32.8)	2.0	1.2-3.3	0.007
*II. Medical history*										
Regular dizziness, n (%)	47 (36.4)	50 (9.1)	5.8	3.6-9.1	<0.001	28 (38.4)	41 (9.3)	6.0	3.4-10.7	<0.001
No. major chronic diseases (0–7), mean (SD)	1.6 (1.1)	1.1 (1.0)	1.4	1.2-1.7	<0.001	1.4 (1.1)	1.1 (1.0)	1.3	1.0-1.5	0.015
Chronic pulmonary disease, n (%)	18 (14.0)	73 (13.2)	1.1	0.6-1.9	0.827	9 (12.3)	58 (13.2)	0.9	0.4-2.0	0.836
Cardiac disease, n (%)	38 (29.5)	134 (24.3)	1.3	0.9-2.0	0.223	19 (26.0)	103 (23.5)	1.1	0.7-2.0	0.634
Peripheral arterial disease, n (%)	14 (10.9)	33 (6.0)	1.9	1.0-3.7	0.053	4 (5.5)	27 (6.2)	0.9	0.3-2.6	0.824
Diabetes mellitus, n (%)	10 (7.8)	36 (6.5)	1.2	0.6-2.5	0.617	5 (6.8)	22 (5.0)	1.4	0.5-3.8	0.517
Stroke, n (%)	5 (3.9)	26 (4.7)	0.8	0.3-2.2	0.683	3 (4.1)	15 (3.4)	1.2	0.3-4.3	0.766
Osteo/rheumatoid arthritis, n (%)	93 (72.1)	248 (44.9)	3.2	2.1-4.8	<0.001	45 (61.6)	216 (49.2)	1.7	1.0-2.8	0.051
Cancer, n (%)	25 (19.4)	58 (10.5)	2.0	1.2-3.4	0.006	14 (19.2)	52 (11.8)	1.8	0.9-3.4	0.087
*III. Medication*										
No. of drugs, mean (SD)	2.9 (2.1)	2.2 (2.0)	1.2	1.1-1.3	0.001	2.5 (2.0)	2.0 (1.9)	1.1	1.0-1.3	0.030
Psychopharmaceutical drugs										
Antidepressants, n (%)	8 (6.2)	20 (3.6)	1.8	0.8-4.1	0.189	2 (2.7)	16 (3.6)	0.7	0.2-3.3	0.698
Anxiolytics, n (%)	13 (10.1)	28 (5.1)	2.1	1.1-4.2	0.035	9 (12.3)	20 (4.6)	2.9	1.3-6.8	0.011
Hypnotics, n (%)	19 (14.7)	51 (9.2)	1.7	1.0-3.0	0.067	8 (11.0)	37 (8.4)	1.3	0.6-3.0	0.481
Cardiovascular drugs										
Antihypertensives, n (%)	53 (41.1)	191 (34.6)	1.3	0.9-2.0	0.168	32 (43.8)	147 (33.5)	1.6	0.9-2.6	0.086
Diuretics, n (%)	28 (21.7)	75 (13.6)	1.8	1.1-2.9	0.022	12 (16.4)	49 (11.2)	1.6	0.8-3.1	0.201
Nitrates, n (%)	19 (14.7)	32 (5.8)	2.8	1.5-5.1	0.001	9 (12.3)	27 (6.2)	2.1	1.0-4.8	0.061
*IV. Psychological*										
Impaired cognition, MMSE ≤24, n (%)	9 (7.0)	25 (4.5)	1.6	0.7-3.5	0.254	4 (5.5)	15 (3.4)	1.6	0.5-5.1	0.392
Anxiety, HADS-A ≥11, n (%)^a,f^	17 (13.3)	17 (3.1)	4.8	2.4-9.7	<0.001	7 (9.6)	13 (3.0)	3.5	1.4-9.0	0.011
Depression, CES-D ≥16, n (%)^a,f^	40 (31.3)	78 (14.2)	2.8	1.8-4.3	<0.001	20 (27.4)	68 (15.5)	2.1	1.2-3.7	0.014
Anxiety or depression ^a,f^	43 (33.6)	83 (15.1)	2.9	1.8-4.4	<0.001	21 (28.8)	72 (16.4)	2.1	1.2-3.6	0.013
*V. Sensory*										
Impaired vision, n (%)^b^	42 (32.6)	94 (17.1)	2.3	1.5-3.6	<0.001	20 (27.4)	71 (16.2)	2.0	1.1-3.5	0.022
Impaired hearing, n (%)	49 (38.0)	169 (30.6)	1.4	0.9-2.1	0.107	26 (35.6)	127 (28.9)	1.4	0.8-2.3	0.249
*VI. Balance/gait*										
Impaired function of lower extremities (gait speed), n (%)^c,g^	52 (41.3)	130 (23.8)	2.3	1.5-3.4	<0.001	25 (34.2)	98 (22.6)	1.8	1.0-3.0	0.033
Impaired function of lower extremities (chair stands), n (%)^d,h^	65 (51.2)	146 (26.5)	2.9	2.0-4.3	<0.001	34 (47.2)	108 (24.7)	2.7	1.6-4.6	<0.001
Impaired standing balance (tandem stand), n (%)^e,g^	35 (27.8)	97 (17.8)	1.8	1.1-2.8	0.012	17 (23.3)	68 (15.7)	1.6	0.9-3.0	0.110
Falling past year, n (%)^a,f^	46 (35.7)	159 (28.9)	1.4	0.9-2.0	0.134	25 (34.2)	128 (29.2)	1.3	0.7-2.1	0.386

CI: Confidence Interval; D+: presence of dizziness; D-: absence of dizziness; OR: Odds Ratio.

Missing values at 7-year follow-up in ^a^0.3%, ^b^0.1%, ^c^1.2%, ^d^0.6%, and ^e^1.6% of respondents.

Missing values at 10-year follow-up in ^f^0.2%, ^g^1.0%, and ^h^0.4% of respondents.

Participants who dropped out at 7- and 10-year follow-up were significantly older (both *P* <0.001), more often male (*P* = 0.004 and 0.04), used more drugs at baseline (both *P* <0.001), and had more major chronic diseases at baseline (both *P* <0.001).

Seven variables (anxiety, depression, impaired vision, gait speed, chair stands, tandem stand, and fall history) had limited missing values (0.1% to 1.6%). When comparing missing values in dizzy and non-dizzy participants at 7- and 10-year follow-up, the number of missing values did not differ significantly.

### Multivariable associations with dizziness

Predictors selected for the model predicting regular dizziness at 7-year follow-up were living alone, history of dizziness, history of osteo/rheumatoid arthritis, history of cancer, use of nitrates, presence of anxiety or depression, impaired vision, and impaired function of lower extremities as measured by chair stands (Table 2). Predictors selected for the model predicting regular dizziness at 10-year follow-up were living alone, history of dizziness, history of cancer, use of anxiolytics, and impaired function of lower extremities.

**Table 2:** Predictors of regular dizziness in an older community population at 7- and 10-year follow-up*

Characteristic	B	SE	Wald	OR	95% CI	*P*value
*7-year follow-up* ^a^						
Living alone	0.450	0.229	3.8	1.6	1.0-2.5	0.050
History of dizziness	1.324	0.265	25.0	3.8	2.2-6.3	<0.001
History of osteo/rheumatoid arthritis	0.644	0.239	7.3	1.9	1.2-3.0	0.007
History of cancer	0.524	0.307	2.9	1.7	0.9-3.1	0.088
Use of nitrates	0.835	0.351	5.6	2.3	1.2-4.6	0.018
Presence of anxiety or depression	0.541	0.262	4.3	1.7	1.0-2.9	0.039
Impaired vision	0.611	0.254	5.8	1.8	1.1-3.0	0.016
Impaired function of lower extremities (chair stands)	0.670	0.229	8.6	2.0	1.2-3.1	0.003
*10-year follow-up* ^b^						
Living alone	0.460	0.278	2.7	1.6	0.9-2.7	0.098
History of dizziness	1.555	0.306	25.9	4.7	2.6-8.6	<0.001
History of cancer	0.552	0.357	2.4	1.7	0.9-3.5	0.122
Use of anxiolytics	0.861	0.475	3.3	2.4	0.9-6.0	0.070
Impaired function of lower extremities (chair stands)	0.744	0.281	7.0	2.1	1.2-3.7	0.008

CI: Confidence Interval; OR: Odds Ratio; SE: Standard Error.

*Multivariable logistic regression analysis, using backward elimination (Wald test) with a *P* value of 0.157 used for removal.

^a^AUC 0.78 [0.73-0.82], adjusted AUC 0.77 [0.75-0.78], Hosmer-Lemeshow 0.36.

^b^AUC 0.72 [0.65-0.78], adjusted AUC 0.71 [0.68-0.72], Hosmer-Lemeshow 0.31.

When excluding history of dizziness as potential predictor, the odds ratios (OR) of all identified predictors slightly increased (see Additional file 1).

### Performance of the models

According to the Hosmer-Lemeshow statistic, the reliability of the models was adequate (*P* value of 0.36 for 7-year follow-up and 0.31 for 10-year follow-up). Calibration of the 7-year follow-up model was good, with the slope of the calibration plot approaching the diagonal (see Additional file 2). The slope of the 10-year follow-up calibration plot was below 1, indicating optimism of the model. The discriminative ability of the 7-year and 10-year model was acceptable (AUC 0.78 [0.73-0.82] and 0.72 [0.65-0.78], also after adjustment for over-optimism (adjusted AUC 0.77 [0.75-0.78] and 0.71 [0.68-0.72]).

When excluding history of dizziness as potential predictor, the discriminative ability of the 7-year and 10-year model decreased (from 0.78 to 0.75 and from 0.72 to 0.68, respectively; see Additional file 1).

## Discussion

In this study we investigated long-term predictors of dizziness in a prospective cohort study among community-dwelling older persons. We found multiple factors to predict regular dizziness. Living alone, history of dizziness, history of osteoarthritis or rheumatoid arthritis, use of nitrates, presence of anxiety or depression, impaired vision, and impaired function of lower extremities independently predicted regular dizziness at 7-year follow-up. Furthermore, history of dizziness and impaired function of lower extremities predicted regular dizziness at 10-year follow-up.

Until now, most studies investigating characteristics of dizziness in community-dwelling older adults used a cross-sectional study design [1, 3–5, 16, 17]. Only two studies performed a prospective cohort study to investigate predictors of dizziness. Gassmann et al. studied a sample of 620 persons aged 65+ years in a 2-year follow-up study [2] and Olsson Möller et al. studied 1,273 persons aged 60+ years during 3- and 6-year follow-up [26]. Although both studies provide essential data on dizziness in older adults, these studies were limited by respectively lack of adjustment for gender and age [2], and high percentages of missing values [26].

With the present study we confirm three previously identified predictors of dizziness, i.e. living alone [2], history of dizziness [26], and depression [2]. We also demonstrate previously described associations (with dizziness) to be long-term predictors of dizziness, namely arthritis [17], anxiety [5], depression [3–6, 16], and visual impairment [3, 17]. Contrary to Gassmann et al. [2] we did not identify a long-term relationship between cardiovascular disease and dizziness: history of cardiac disease, peripheral arterial disease, or stroke did not predict dizziness at 7- or 10-year follow-up. This may be due to our much longer follow-up (7 and 10 years versus 2 years) in which more persons dropped out because of severe cardiovascular illness or death (survivor effect). Apart from that, the identified relationship between cardiovascular disease and dizziness by Gassmann et al. was only modest (OR 1.43, 95% CI 0.98-2.09).

Our study is the first to identify impaired function of the lower extremities as an important predictor of regular dizziness in older adults, at 7- as well as at 10-year follow-up. Until now, only one study included physical functioning tests to predict dizziness in older adults [26]. In that study, Ollson Möller et al. investigated the Romberg test and grip strength and found reduced grip strength to predict dizziness in subjects below 80 years of age.

Strengths of our study are the long-term follow-up period, the large representative sample, and the low percentage of missing values. A limitation of our study may be the choice of one prediction model for all age groups. However, a differentiation of age groups (e.g., 60–70, 70–80, 80+) would reduce the number of cases per group, subsequently decreasing the maximum permitted number of candidate predictors and leaving no space for all relevant predictors [28]. Another limitation may be the use of dizziness as an umbrella term, neglecting the four dizziness subtypes vertigo, presyncope, disequilibrium, and other dizziness. However, we believe that differentiating dizziness subtypes is less important in older people, as the majority of older dizzy persons in the community have more than one dizziness subtype [5].

Although our study was not designed to investigate causality, the identified predictors may provide important leads for the management of dizziness in older persons. Examples are the use of nitrates (medication adjustment), presence of anxiety or depression (psychotherapy), visual impairment (correction of vision), and impaired function of lower extremities (physiotherapy). Treating these characteristics in dizzy older patients may reduce not only symptoms of dizziness but also dizziness-related impairment [29]. To investigate the effectiveness of such a multifactorial targeted intervention, we are performing a randomized controlled clinical trial among older dizzy patients in general practice (trial number NTR4346).

From a wider perspective, we believe our study adds important evidence to the concept of dizziness as a geriatric syndrome. Geriatric syndromes are multifactorial health conditions that occur when the accumulated effect of impairments in multiple systems renders a person vulnerable to situational challenges [5]. Sloane and Dallara already pointed at the limitations of the current diagnosis-oriented approach of dizziness and expressed the need for strategies that more effectively reduce symptoms and dizziness-related disability [30]. Tinetti et al. suggested that considering dizziness a geriatric syndrome might lay the groundwork for such an impairment reduction strategy [5]. Although other research groups support the concept of dizziness as a geriatric syndrome [3, 4, 16, 17], the scientific evidence is limited due to cross-sectional study designs. With the present study we strengthen the case for the concept of dizziness as a geriatric syndrome, by demonstrating the long-term relationship between dizziness and multiple predisposing characteristics. Hopefully, our results contribute to a necessary shift of paradigm: when evaluating dizziness in older patients, clinicians should not just focus on diagnosing underlying diseases but also on identifying contributing factors that are potentially modifiable.

## Conclusions

Multiple factors predict regular dizziness in community-dwelling older adults. A multifactorial approach – targeting potentially modifiable predictors – may add to the current diagnosis-oriented approach of dizziness in older patients.

## Electronic supplementary material

Additional file 1: **Predictors of regular dizziness in an older community population at 7- and 10-year follow-up – Additional analysis: without ‘history of dizziness’ as potential predictor.** (PDF 17 KB)

Additional file 2: **Calibration plots for 7- and 10-year follow-up.** (PDF 33 KB)

## Abbreviations

AUC: Area under the receiver operating characteristic curve
CES-D: Center for Epidemiological Studies Depression scale
CI: Confidence interval
GP: General practitioner
HADS: Hospital Anxiety and Depression Scale
LASA: The Longitudinal Aging Study Amsterdam
MMSE: Mini-Mental State Examination
OR: Odds ratio
SE: Standard error.
