# Supplementary material for: Predictors of dizziness in older persons: a 10-year prospective cohort study in the community
Source: BMC Geriatr. 2014 Dec 15;14:133. doi: 10.1186/1471-2318-14-133 (PMC4274723; doi:10.1186/1471-2318-14-133)
Supplement: Supplementary file 1 — Additional file 1: Predictors of regular dizziness in an older community population at 7- and 10-year follow-up – Additional analysis: without ‘history of dizziness’ as potential predictor. (PDF 17 KB) [file 12877_2014_1066_MOESM1_ESM.pdf]

**Additional file 1. Predictors of regular dizziness in an older community population at 7- and 10-year follow-up – Additional analysis: *without* 'history of dizziness' as potential predictor**

| Characteristic | B | SE | Wald | OR | 95% CI | *P* value |
|---|---|---|---|---|---|---|
| *7-year follow-up* [a] | | | | | | |
| Years of education | -0.066 | 0.036 | 3.3 | 0.94 | 0.9-1.0 | 0.069 |
| Living alone | 0.422 | 0.223 | 3.6 | 1.5 | 0.9-2.4 | 0.058 |
| History of osteo/rheumatoid arthritis | 0.762 | 0.233 | 10.7 | 2.1 | 1.4-3.4 | 0.001 |
| Use of nitrates | 0.879 | 0.342 | 6.6 | 2.4 | 1.2-4.7 | 0.010 |
| Presence of anxiety or depression | 0.735 | 0.247 | 8.9 | 2.1 | 1.3-3.4 | 0.003 |
| Impaired vision | 0.698 | 0.245 | 8.1 | 2.0 | 1.2-3.2 | 0.004 |
| Impaired function of lower extremities (chair stands) | 0.704 | 0.222 | 10.0 | 2.0 | 1.3-3.1 | 0.002 |
| *10-year follow-up* [b] | | | | | | |
| Living alone | 0.466 | 0.269 | 3.0 | 1.6 | 0.9-2.7 | 0.083 |
| History of cancer | 0.568 | 0.348 | 2.7 | 1.8 | 0.9-3.5 | 0.103 |
| Use of anxiolytics | 0.902 | 0.453 | 4.0 | 2.5 | 1.0-6.0 | 0.046 |
| Impaired vision | 0.497 | 0.314 | 2.5 | 1.6 | 0.9-3.0 | 0.114 |
| Impaired function of lower extremities (chair stands) | 0.894 | 0.271 | 10.9 | 2.4 | 1.5-4.2 | 0.001 |

CI: Confidence Interval; OR: Odds Ratio; SE: Standard Error.

*: Multivariable logistic regression analysis, using backward elimination (Wald test) with a *P* value of 0.157 used for removal.

[a]: AUC 0.75 [0.70-0.80].

[b]: AUC 0.68 [0.61-0.75].
