# Supplementary figures and images for: Predictors of dizziness in older persons: a 10-year prospective cohort study in the community
Source: BMC Geriatr. 2014 Dec 15;14:133. doi: 10.1186/1471-2318-14-133 (PMC4274723; doi:10.1186/1471-2318-14-133)

## Additional file 2. Calibration plots for 7- and 10-year follow-up

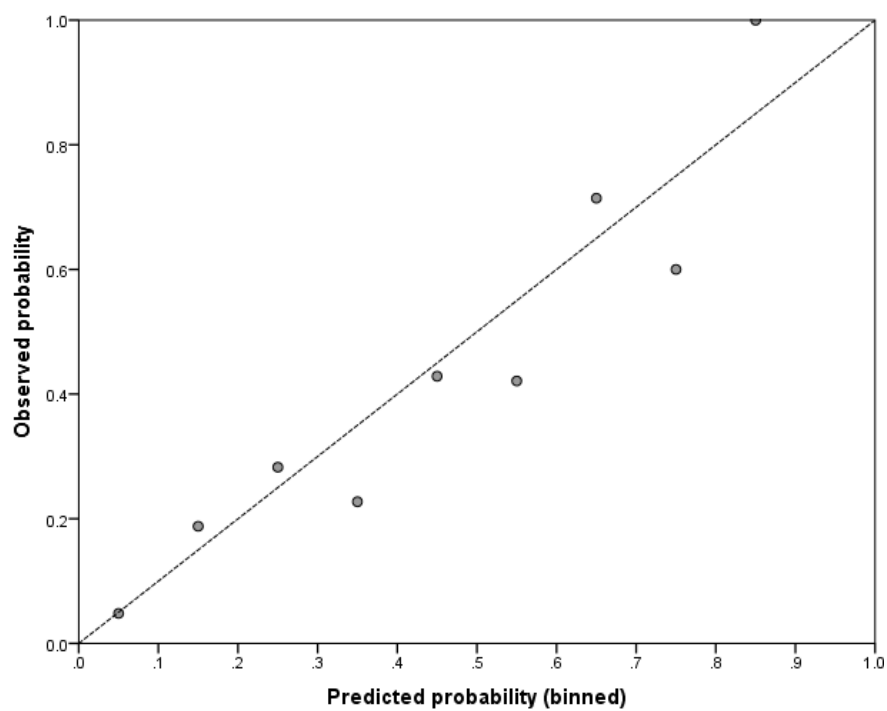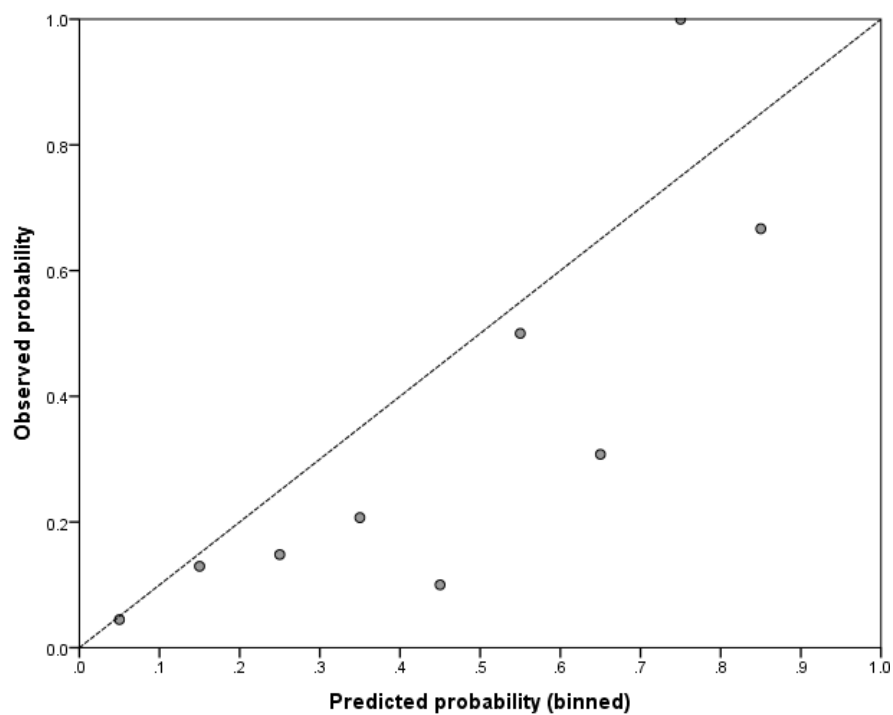

Supplement: Supplementary file 2 — Additional file 2: Calibration plots for 7- and 10-year follow-up. (PDF 33 KB) [file 12877_2014_1066_MOESM2_ESM.pdf]
